# Supplementary material for: Effects of UVC Treatment on Biofilms of Escherichia coli Strains Formed at Different Temperatures and Maturation Periods
Source: Foods. 2025 Sep 3;14(17):3091. doi: 10.3390/foods14173091 (PMC12428618; doi:10.3390/foods14173091)
Supplement: Supplementary file 1 [file foods-14-03091-s001.zip › foods-3699547-supplementary.pdf]

foods

MDPI

Table S1. Morphological, biochemical characteristics, and API test identification of colonies[1].

	Colony color in MacConkey agar	Tests Results																				API identification (%ID)
		ONPG	ADH	LDC	ODC	CIT	H_2S	URE	TDA	IND	VP	GEL	GLU	MAN	INO	SOR	RHA	SAC	MEL	AMY	ARA	
Colony 1	Pink	+	-	+	+	-	-	-	-	+	-	-	+	+	-	+	+	-	+	-	+	*Escherichia coli* (99.9%)
Colony 2	Pink	+	-	+	-	+	-	+	-	-	+	-	-	+	+	+	+	+	+	+	+	*Klebsiella pneumoniae* ssp. pneumoniae (97.3%)
Colony 3	Pink	+	+	-	+	+	-	-	-	-	+	-	-	+	-	+	+	+	+	+	+	*Enterobacter cloacae* (98.6%)
Colony 4	Colorless	+	+	-	-	+	-	-	-	+	-	+	-	+	-	-	-	+	+	+	+	*Aeromonas hydrophila/caviae/sobria* (94.9%)
Colony 5	Colorless	+	-	-	-	+	-	-	-	-	+	+	+	+	+	+	+	+	+	+	+	*Serratia ficaria* (97.1%)

[1]ONPG: 2-nitrophenyl-ßD-galactopyranoside; ADH: L-arginine; LDC: L-lysine; ODC: L-ornithine; CIT: trisodium citrate; H_2S: sodium thiosulfate; URE: urea; TDA: L-tryptophane; IND: L-tryptophane; VP: sodium pyruvate; GEL: gelatin (bovine origin); GLU: D-glucose; MAN: D-mannitol; INO: inositol; SOR: D-sorbitol; RHA: L-rhamnose; SAC: D-sucrose; MEL: D-melibiose; AMY: amygdalin; ARA: L-arabinose.
